# Traumatic knee injury healthcare pathways and outcomes: the Australian Knee Injury Inception Cohort Study (KIICS) protocol

**DOI:** 10.1136/bmjsem-2025-002983

**Published:** 2025-11-27

**Authors:** Marc-Olivier Dubé, Kay M Crossley, Andrea M Bruder, Brooke E Patterson, Sean Kaplan, Melissa J Haberfield, Christian J Barton, Stephanie R Filbay, Michelle M Dowsey, Sean I Docking, Joshua R Zadro, Iliana N Ackerman, Joanna Kvist, Evangelos Pappas, Tenille Moselen, Adam G Culvenor

**Affiliations:** 1 La Trobe Sport and Exercise Medicine Research Centre, La Trobe University, Melbourne, Victoria, Australia; 2 Australian International Olympic Committee (IOC) Research Centre, La Trobe University, Melbourne, Victoria, Australia; 3 Department of Physiotherapy, Podiatry, Prosthetics and Orthotics, La Trobe University, Melbourne, Victoria, Australia; 4 Centre for Health, Exercise and Sports Medicine, University of Melbourne, Melbourne, Victoria, Australia; 5 Department of Surgery, University of Melbourne, Melbourne, Victoria, Australia; 6 Musculoskeletal Health and Wiser Health Care Units, Monash University, Melbourne, Victoria, Australia; 7 Sydney Musculoskeletal Health, The University of Sydney and Sydney Local Health District, Sydney, New South Wales, Australia; 8 Department of Health, Medicine and Caring Sciences, Linköping University, Linkoping, Sweden; 9 School of Health and Biomedical Sciences, Royal Melbourne Institute of Technology, Melbourne, Victoria, Australia; 10 Sydney School of Health Sciences, The University of Sydney, Sydney, New South Wales, Australia

**Keywords:** Anterior cruciate ligament, Surgery, Rehabilitation, Sports medicine

## Abstract

Decision-making for the optimal management of traumatic knee injuries can be challenging. Clinical trials reveal only small differences between surgical and non-surgical approaches, while patient and clinician biases, as well as healthcare access issues, may also influence management. Little is known about the real-world healthcare pathways for patients with a knee injury, as well as the person- and/or healthcare-related factors that influence management strategies and outcomes. The Australian Knee Injury Inception Cohort Study (KIICS) aims to: (1) describe healthcare pathways following acute knee injury (including the timing and type of healthcare consultations); (2) identify patient- and/or healthcare-related predictors of management strategy (ie, surgical vs non-surgical); and (3) examine the long-term outcome of different injury types, healthcare pathways and management strategies. KIICS is a nationwide prospective longitudinal inception cohort study recruiting Australians who have sustained an acute knee injury within the previous 6 months that disrupted daily activities or sports and led to a healthcare consultation. Participants will complete online questionnaires at enrolment and at 6 months, 1, 2, 5 and 10 years post-injury. The data to be collected will include sociodemographic characteristics, knee injury history, the sequence of healthcare consultations and referral patterns, and management strategies (ie, surgical vs non-surgical). Patient-reported outcomes will include knee pain and instability, knee-related quality of life, patient-acceptable symptom state, health-related quality of life, mental health, fear of reinjury, return-to-sport status and activity level. Detailed statistical analysis plans will be developed to address the study’s key research questions, informing clinical practice, shared decision-making and healthcare policy.

**WHAT IS ALREADY KNOWN ON THIS TOPIC**

- Rates of traumatic knee injury and related surgery are rising globally.
- Rehabilitation with the option for surgery later, if required, leads to similar outcomes when compared with early surgery plus rehabilitation for common traumatic knee injuries such as acute anterior cruciate ligament rupture and meniscal tear.

**WHAT THIS STUDY ADDS**

- First nationwide inception cohort study of traumatic knee injuries to capture the sequence of healthcare interactions and management decision-making (ie, healthcare pathway leading to surgical or non-surgical management).
- It will identify key factors (patient- and healthcare-related) influencing healthcare pathway and management strategy (ie, surgical or non-surgical management).
- It will provide 5- and 10-year follow-up data to assess long-term outcomes of various traumatic knee injuries, healthcare pathways and management strategies.

**HOW THIS STUDY MIGHT AFFECT RESEARCH, PRACTICE OR POLICY**

- Findings will provide important data as to the multidisciplinary nature of care provided (or lack thereof) and how well care aligns with current best-practice guidelines.
- Data may influence referral pathway behaviour for optimal care and long-term outcomes for knee injuries, such as anterior cruciate ligament ruptures and meniscal tears.
- Results may lay the foundation for establishing a nationwide knee injury registry to comprehensively monitor knee injury outcomes and service provision, and inform future clinical guidelines for knee injury management.

## Introduction

 Rates of traumatic knee injuries, particularly ACL and meniscal tears, are rising globally.^1 2^ Australia has one of the world’s highest and fastest growing rates of ACL injuries and surgical ACL reconstruction (ACLR)—with ACLR rates rising more than 43% since 2000. An estimated 90% of young Australian adults who sustain an ACL injury undergo surgery.^3^ The highest rates of ACLR are seen in 20–24-year-old males at 283 procedures per 100 000 population.^4^ While ACL injuries are the focus of much research, other traumatic knee injuries, such as meniscal tears, medial and lateral collateral ligament (MCL, LCL) injuries, patellar dislocations and fractures, may also contribute significantly to long-term knee dysfunction and osteoarthritis (OA).

Despite advances in surgical and rehabilitation approaches, long-term outcomes following ACL and meniscal injuries remain suboptimal. 45% of patients do not return to their pre-injury level of sport.^5^ One in six and one in seven report persistent symptoms (eg, pain) up to 6 years post-ACLR^6^ and up to 2 years post-arthroscopic meniscus repair,^7^ respectively, contributing to reduced quality of life.^8^ On average, patients with a previous traumatic knee injury report worse knee health than their uninjured peers many years after their initial injury.^9^ In the long term, traumatic knee injuries confer a fourfold to sixfold increased risk of knee OA,^10^ and progression to total knee replacement is relatively quick, with sports-related knee injury more than doubling the hazard of subsequent knee replacement over 15 years.^11^ Given the rising incidence of traumatic knee injuries among young, active individuals and their long-term burden, these injuries represent an emerging public health concern with implications for both individual well-being and healthcare system sustainability.^12^

For ACL and meniscal tears, selecting the optimal management strategy can be challenging, particularly given longstanding misconceptions. Indeed, many patients^13^ believe that surgery is necessary for optimal recovery and return to sport, as well as reducing the risk of developing knee OA, which may be due to external influences (eg, clinicians,^14^ media). However, randomised controlled trials (RCTs) have challenged this belief, showing that long-term outcomes are largely similar following early surgery plus rehabilitation compared with rehabilitation alone, with the option of later surgery.^15^,^17^ Uptake of non-surgical, evidence-based rehabilitation remains low—likely due to entrenched beliefs, lack of access and systemic barriers.^18^ Choosing ACLR as a first-line intervention for all may not be cost-effective.^19^ The direct costs of ACL surgery and rehabilitation in Australia are ~$A15 000 per person, and projected to exceed $A314 million annually by 2030.^20^ Understanding the factors that influence patients’ uptake of different management strategies and how they navigate the healthcare system is essential to inform strategies to assist decision-making.

Little is known about what drives the healthcare pathways taken by individuals with acute knee injuries, including which providers they see, in what order, and how these sequences influence management strategy (eg, surgical vs non-surgical). Research on other musculoskeletal conditions, such as low back pain, suggests that the initial healthcare encounter and referral patterns significantly impact the quality of care and patient health outcomes.^21^ Similar patterns may exist in knee injury care, with current evidence indicating large variations in healthcare pathways for ACL injuries in the UK.^22^ Large-scale registries, such as those in Scandinavia,^23 24^ have provided valuable insights into ACL injury management, including predictors of clinical outcomes and graft failure. However, these registries focus on surgically managed patients, and no equivalent registry or national longitudinal cohort exists in Australia. Real-world longitudinal data comparing surgical and non-surgical pathways outside the context of RCTs are lacking in Australia, although such research has been conducted in other countries.^25^ It is critical to understand the effectiveness of various management strategies, including multidisciplinary or stepped care pathways.

**Figure 1:**
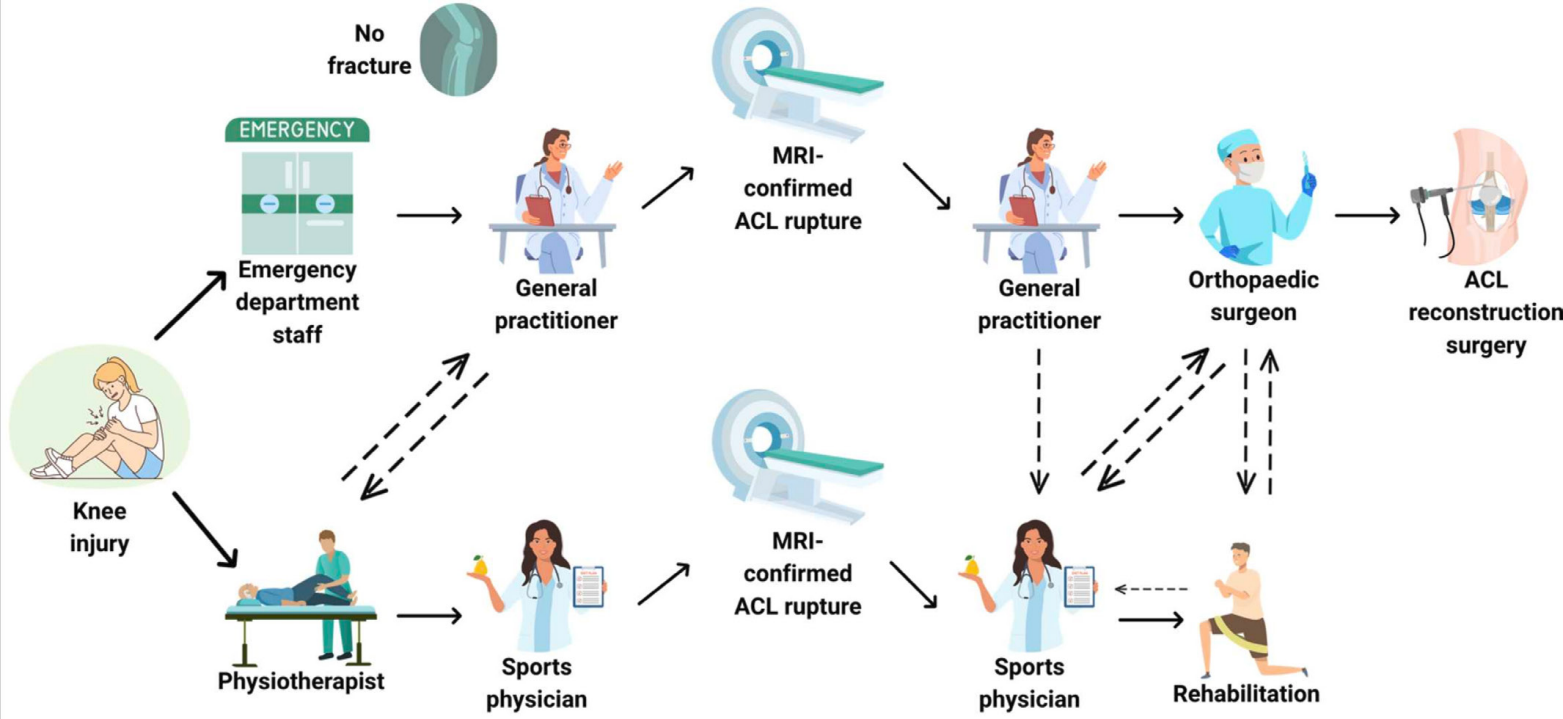
Examples of healthcare pathways for Australians sustaining a knee injury.

**Figure 2:**
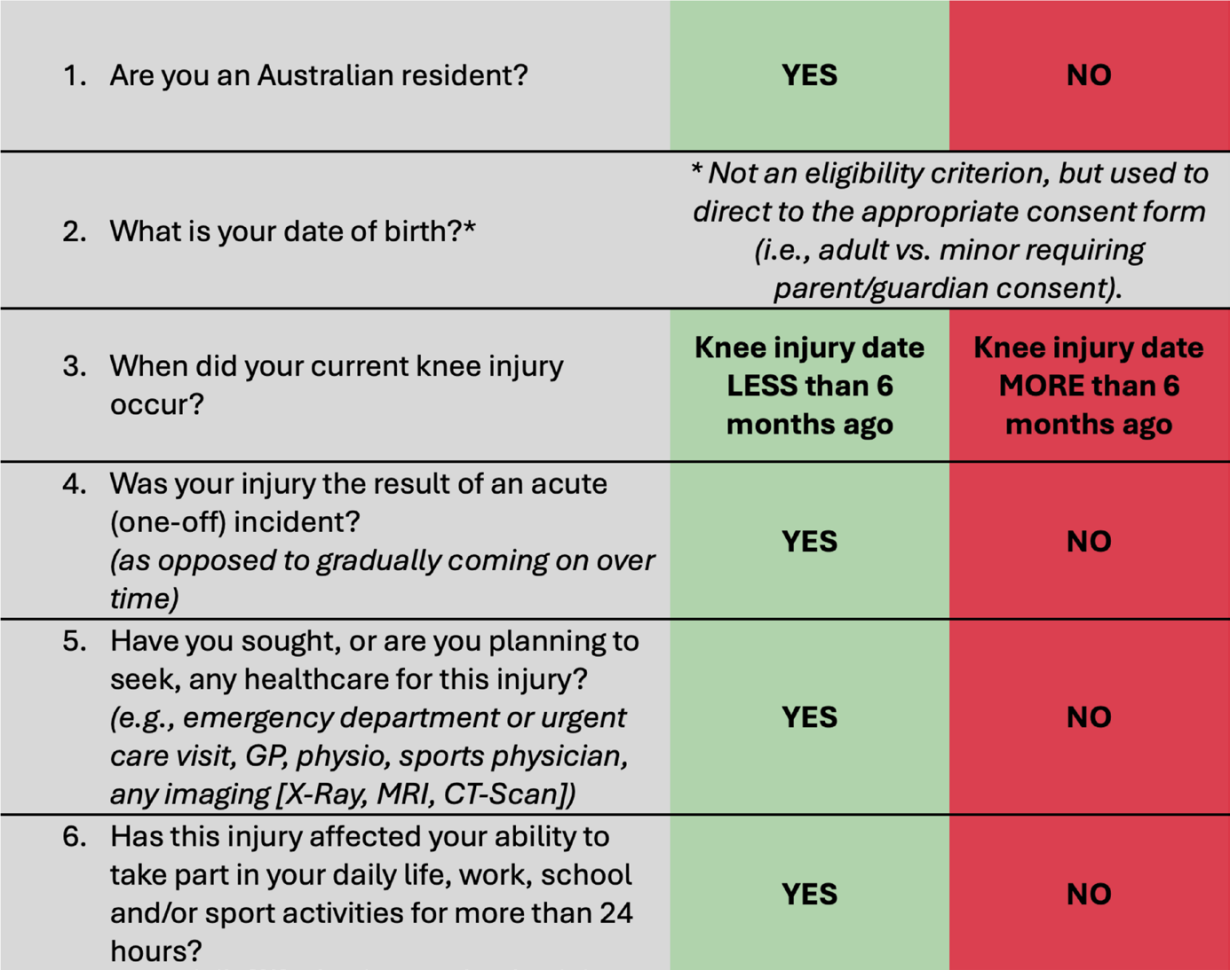
Eligibility screening questions. GP, general practitioner.

The Australian Knee Injury Inception Cohort Study (KIICS) aims to: 1) describe healthcare pathways following acute knee injury (including the timing and type of healthcare consultations) (figure 1); 2) identify patient- and/or healthcare-related predictors of management strategy (ie, surgical vs non-surgical); 3) examine the long-term outcome of different injury types, healthcare pathways and management strategies.

## Methods and analysis

### Study design

This study is a nationwide longitudinal prospective inception cohort study that will recruit Australian residents who sustained a self-reported traumatic knee injury in the previous 6 months. Data will be collected online through the Research Electronic Data Capture (REDCap; Vanderbilt University, Tennessee, USA) web-based survey system hosted on a secure server at La Trobe University.

### Participants

Australian residents of any age who have sustained a traumatic knee injury in the previous 6 months are eligible. A traumatic knee injury is defined as a knee injury of sudden onset that is severe enough to interrupt daily life, work, school and/or sport activities for more than 24 hours, resulting in contact with the healthcare system (eg, for imaging, diagnosis and/or management). Australian residents are defined as those living in Australia at the time of their knee injury and throughout most of their healthcare pathway. The aim is to recruit at least 1000 participants.

### Recruitment procedure

Recruitment commenced in August 2024 and will continue until 2027. Enrolment will occur nationwide, on an opt-in basis. Recruitment initially commenced in community settings, with subsequent involvement from public hospitals that will recruit from public healthcare settings (eg, emergency department, outpatient orthopaedic/physiotherapy clinics).

In community settings, participants will be recruited through: direct referral from treating healthcare providers; posters and flyers at healthcare clinics (eg, sports medicine, physiotherapy, general practitioner, radiology, orthopaedic) and community organisations (eg, sports clubs, gyms, universities) who agreed to promote the study; posters and flyers on social media, community notice boards and community newsletters.

In public healthcare settings, participants will be recruited through: direct referral from treating healthcare providers via study invitation sent to patients who were treated for an acute knee injury in the past 6 months; posters and flyers in emergency department waiting rooms and relevant outpatient clinics.

All recruitment materials will include a weblink and QR code to the study home page (www.kneeinjurystudy.com.au), where potential participants can register their interest, provide consent and complete the screening and baseline questionnaires if deemed eligible. Individuals interested in participating in the study will be automatically screened for eligibility within REDCap by answering six questions (figure 2). Eligible participants will be directed to the online patient information and consent form. Once participants have provided consent (via e-signature), they will be directed to the baseline questionnaire. For participants under 18 years of age, parental/guardian consent (e-signature) will be required.

### Data collection procedure

Data will be collected at baseline (on initial enrolment in the study) and at 6 months, 1, 2, 5 and 10 years after the initial injury. For each follow-up, participants will receive an email with a link to the online questionnaire. To support retention and address potential issues, such as participants changing their email addresses during the study, reminders will be sent via email, phone call and text message. Informed by our consumer advisory group (CAG), data collection will be kept to a minimum at each time point (less than 5–10 min) to minimise participant burden and encourage retention. For this reason, some questionnaires include only specific subscales and single-item scores for certain constructs (online supplemental table 1) provides detailed information about the questionnaires. If any questionnaire is incomplete or further clarification is required regarding participant responses, a member of the research team will telephone participants to follow-up or seek clarification.

### Outcomes

#### Baseline demographic, injury characteristics and outcome expectations

Demographic characteristics will include age, sex, gender and current residential postcode. The postcode will be used to classify geographic location (eg, metropolitan vs regional, state of residence) using the Australian Statistical Geography Standard and infer socioeconomic status (via linkage with the Socio-Economic Indexes for Areas index). Details regarding current knee injury will include the involved side (index knee), the Australian state and setting (eg, sport, work, other) where the injury occurred, diagnosis and how it was confirmed (eg, self-reported, clinician-diagnosed or imaging-confirmed), as well as the patient’s patient preference for management options. For those who have not undergone post-injury surgery at baseline, management preference will be assessed with the question ‘If you were asked right now to choose surgery or rehabilitation without surgery, which option would most accurately represent your answer?’ with five response options ranging from ‘Strong preference for surgery’ to ‘Strong preference for rehabilitation without surgery’. In addition to self-report, knee injury diagnosis will be confirmed via imaging reports (if imaging has been performed) from clinical services. With participant consent, a member of the research team will contact the clinic or imaging provider where the participant reported having their imaging performed, to request a copy of the report. Information about prior knee injuries (eg, side involved, diagnosis) will also be recorded. Participants’ recovery expectations will also be assessed with the question ‘Overall, how do you expect your knee to be 1 year after your injury?*’* with responses recorded on a 7-point Likert scale (range: 1=very much worse, 7=completely recovered) which corresponds to the Global Rating of Change scale used to assess self-perceived change in knee status (table 1).

**Table 1:** Overview of outcomes

	Baseline	6 months	1 year	2 years	5 years	10 years
Demographic and knee injury characteristics						
Age, sex, gender, postcode	√					
Current and prior knee injury details	√					
Recovery expectations	√					
Self-reported outcomes						
Healthcare pathway Private insurance cover and how treatment was paid for Healthcare provider seen, sequence and timing Referral patterns Imaging acquisition Surgical procedures Rehabilitation participation		√	√			
Patient acceptable symptom state	√	√	√	√	√	√
Health-related quality of life (EQ-5D-5L)	√	√	√	√	√	√
Mental health (100-point VAS)	√	√	√	√	√	√
Knee pain (100-point VAS)	√	√	√	√	√	√
Knee instability (100-point VAS)	√	√	√	√	√	√
Activity level (TAS)	√	√	√	√	√	√
New/reinjury occurrence and details		√	√	√	√	√
Global rating of change		√	√	√	√	√
Function in sport and recreation (KOOS-Sport/Rec)		√	√	√	√	√
Knee-related quality of life (KOOS-QoL)		√	√	√	√	√
Fear of reinjury (100-point VAS)		√	√	√	√	√
Surgery occurrence and details				√	√	√

KOOS, Knee injury and Osteoarthritis Outcome Score; TAS, Tegner Activity Scale; VAS, visual analogue scale.

#### Healthcare pathway

Each participant’s healthcare pathway following knee injury will be documented via self-report regarding:

- Use of private health insurance vs public healthcare, and how knee injury treatments (eg, surgery, rehabilitation) were funded;
  - Private health insurance paid some/all of the treatment cost; sports injury insurance covered some/all of the treatment cost; my employer/work cover (worker compensation insurance) covered some/all of the treatment cost; I paid all treatment costs out of pocket (no insurance); treatment was provided through the public health system at no/little cost to me; I don’t remember; other (if other, free text to provide details).
- Every type of unique healthcare provider consulted for their knee injury, in what sequence and time post-injury consulted;
  - Type: emergency department staff, general practitioner, sports physician, physiotherapist, orthopaedic surgeon, other (if other, free text to provide details).
  - Sequence: First, second, third, fourth, fifth healthcare provider consulted
  - Timing: within 1 week post-injury, >1 to 2 weeks post-injury, >2 to 4 weeks post-injury, >1 to 2 months post-injury, >2 to 3 months post-injury, >3 to 6 months post-injury, >6 months post-injury.
- Who referred them (general practitioner, emergency department staff or sports physician if applicable) and where they were referred to (imaging, general practitioner, sports physician, physiotherapist, orthopaedic surgeon, outpatient hospital clinic, other (if other, free text to provide details)).
- Imaging acquisition details (eg, type of imaging (X-ray, MRI, CT, ultrasound) and clinic where it was acquired) and surgical procedure details (eg, type, when, graft used if ACLR) if applicable.
- Rehabilitation professional involvement, and if so, how long (eg, length of follow-up and number of sessions since injury).

#### Patient satisfaction

Patient satisfaction with knee health will be assessed using the patient acceptable symptom state (PASS), which is a single question with a yes/no response: ‘Taking into account your level of pain and function, if you were to remain for the next few months as you are today, would you consider that your current state is satisfactory?’. The PASS is frequently used to assess symptom status in conjunction with traditional patient-reported outcome measures. It has shown good content validity and good to excellent reliability (intra-class correlation coefficient=0.8, online supplemental table 1) in the context of knee injuries.

#### Health-related quality of life

Health-related quality of life will be assessed with the EQ-5D-5L instrument and overall rating of health on a visual analogue scale (EQ-VAS). The instrument covers five domains: mobility, self-care, usual activities, pain or discomfort, and anxiety or depression. The presence and severity of problems on each domain are rated on a 5-point Likert scale (no problems, slight, moderate, severe, extreme problems). Responses are used to generate a health profile with 3125 possible health states defined by combining one level from each dimension, ranging from 11 111 (full health) to 55 555 (worst health).^26^ These health states are converted into a single index ‘utility’ score using an Australian value set, with possible values ranging from −0.30 (worse than death) to 1.0 (perfect health).^27^ The EQ-VAS component asks patients to rate their perceived health today from 0 (worst imaginable health) to 100 (best imaginable health).^26^ While most validation studies of the EQ-5D in knee injury populations have focused on patients with OA, it is widely used in large knee injury registries, including Scandinavian registries, such as the Swedish National Knee Ligament Registry, supporting its utility for comparison across studies and populations.

#### Mental health

Mental health status will be assessed with the question, ‘In the last week, how has your knee injury impacted your mental health?’. Responses will be recorded on a 100-point VAS (0=no impact on mental health, 100=extreme impact on mental health). VAS has demonstrated validity for assessing constructs like pain and emotional distress (online supplemental table 1), and adapting them for mental health outcomes related to knee injuries is an accepted and practical approach.

#### Knee pain and instability

Knee pain will be assessed with the question ‘In the last week, on average, how painful was your knee?’. Responses will be recorded on a 100-point VAS (0=no pain, 100=worst pain). Knee instability is assessed with two questions: (1) ‘During the last week (or since your injury if your injury was less than 1 week ago), how stable is your knee in everyday situations?’; and () ‘During the last week (or since your injury if your injury was less than 1 week ago), how stable is your knee when you do, or try to do, rehabilitation training, recreational activities or sports activities?’. Responses to each instability question will be recorded on a 100-point VAS (0=no instability, 100=worst possible instability).

#### Activity level and return to sport

The level of knee-demanding physical activity will be assessed using the Tegner Activity Scale (TAS) which evaluates activity level on a 0–10 scale, where 0=sick leave or disability pension because of knee problems and 10=participation in knee-demanding competitive sports, such as soccer, football or rugby, at a national or international elite level. The TAS is valid, reliable and responsive to changes in populations with knee injuries including ACL tears (online supplemental table 1). Return to sport will be assessed by comparing participants’ post-injury TAS score to their pre-injury TAS score. Only participants who were active at TAS ≥6 pre-injury will be considered eligible for the return-to-sport analysis. For these participants, we will then assess whether they have indicated returning to level 6 or above on the TAS, and whether the sport they are back to is the same as the one they indicated participating in before their injury on their initial questionnaire.

#### Reinjuries and further surgery

The incidence of subsequent/recurrent injuries to the index knee or contralateral knee will be recorded by asking participants to report all future knee injuries to either knee since enrolment, including the specific diagnosis (if known), whether they subsequently underwent surgery and if so, the type and timing of the surgery.

#### Global rating of change

Self-perceived change in knee status from the time of injury will be assessed with the question ‘Overall, how would you rate your knee compared with when you first injured it?’*.* Responses will be recorded on a 7-point Likert scale ranging from 1 (very much worse) to 7 (completely recovered).

#### Knee-related function in sport/recreation and quality of life

Participants will complete two subscales of the Knee injury and Osteoarthritis Outcome Score (KOOS): (1) knee-related function in sport and recreation and (2) knee-related quality of life. Our CAG identified these specific subscales as the most important ones, as they are particularly relevant and responsive in populations of young, active adults with knee injuries. Each subscale consists of 4 items, rated on a 5-point Likert scale (ranging from ‘no problems’ to ‘extreme problems’). Item scores are summed, transformed and presented as a score from 0 to 100, where 0 indicates extreme problems and 100 indicates no problems. The overall KOOS has good psychometric properties and both subscales are valid in knee-injured populations. They have high test–retest reliability (online supplemental table 1). They are commonly used in clinical practice and research to monitor sports and recreational function, as well as knee-related quality of life, after injury.

#### Fear of reinjury

Fear of knee reinjury will be assessed with a single item from the ACL-QoL questionnaire: ‘How fearful are you of re-injuring your knee?’*.* Responses will be recorded on a 100-point VAS (where 0=no fear at all and 100=extremely fearful).

### Sample size calculation

No sample size has been calculated for the cohort; however, we aim for a minimum of 1000 participants. An important element of this study is understanding the extent of knee injuries in Australia, and we will include as many participants as possible during the active recruitment period. If appropriate, the required sample size will be calculated for specific research questions to ensure the analyses conducted are sufficiently powered. If the necessary resources are available, recruitment may extend beyond the expected endpoint of December 2027. This will facilitate the creation of a larger database, enabling the exploration of additional research questions, the evaluation of quality control markers, model-based budget impact analyses or economic evaluations, and the conduct of real-world target trial emulation studies.^28^

### Statistical analyses

Since this nationwide cohort will provide data over multiple years (up to 10-year follow-up) and cover a range of constructs (eg, demographics, injury and treatment characteristics, healthcare pathway, knee function, quality of life, mental health, return to sport), multiple analyses are planned. Specific statistical analysis plans will be developed for each research question, and these will be refined over time with the input from the relevant personnel. Broadly, descriptive statistics will be used to describe participant demographics, injury characteristics and the healthcare pathway. Regression analysis and other statistical methods will be applied to determine the impact of participant baseline characteristics on management strategy (ie, surgical vs non-surgical) and long-term outcomes, as well as the impact of healthcare pathway and management strategy on long-term outcomes. More specifically, for the three main aims:

- Descriptive statistics and visualisation methods (eg, Sankey diagrams) will be used to map and summarise healthcare pathways. The use of latent class cluster analyses will also be considered if healthcare pathways are found to be heterogeneous. Knee injury types (eg, ACL, meniscal, MCL tears) will be reported separately, allowing for targeted analyses based on injury type.
- Multivariable logistic and multinomial regression models will be used to identify patient- and healthcare-related predictors of management strategy (ie, surgical vs non-surgical).
- Long-term outcomes across injury types and management strategy will be evaluated using linear and generalised linear models, adjusted for relevant confounders and baseline characteristics.

To evaluate the representativeness and generalisability of the study, we will compare the baseline characteristics (eg, age, sex/gender, injury type) of KIICS participants with those of other national injury registries and available Australian population data. We will also monitor and report the number of each injury (eg, ACL, meniscal, etc.) identified during the study recruitment period and assess the demographic characteristics (age, sex/gender) of this group to evaluate any potential recruitment bias. These steps will allow us to determine how our cohort sample compares to the broader population of Australians with traumatic knee injuries.

### Data management

All data will be collected and managed via REDCap web-based software (hosted at La Trobe University), facilitating simultaneous data entry. For data analysis, personal data, including participant names, contact details, date of birth and imaging reports, will be securely stored on the La Trobe University server Research Drive Storage, separately from deidentified (numbered) data. All subsequent study data will be identified by participant number only. Participants will be informed that, if they choose to withdraw from the study, their personal health information already collected will be retained, unless an explicit request to the contrary is made, to ensure that the results of the research project can be properly assessed.

### Patient and public involvement

A CAG comprising eight individuals (mean±SD age: 27±7 years; 5 women and 3 men) with lived experience of an acute knee injury (ACL and/or meniscal tear) was involved in the co-design of this study. This CAG was established using the Patient Engagement in Research framework^29^ and drawing on the experience from other CAGs established within our research centre.^30^ Experienced clinicians were also consulted regarding recruitment strategies and relevant outcomes to include. Ongoing CAG consultation will continue to influence recruitment strategies, research questions of importance and the overall acceptability of the study. Consultation meetings with the CAG will be planned to occur 2–4 times per year to provide insights and feedback on study documentation (eg, recruitment material and strategies) as well as relevant research questions and data interpretation. Members of the CAG have received financial compensation for their time spent contributing to the study design and will continue to receive compensation for their time in future meetings.

## Discussion

The healthcare pathways used by patients following traumatic knee injuries remain poorly understood, and the factors (eg, patient- and healthcare-related) influencing management strategy (surgical vs non-surgical) are not well documented in Australia. While evidence from RCTs supports non-surgical management for some people with ACL and meniscal tears, there is a need for complementary real-world longitudinal data to support decision-making in clinical practice. This study will be the first nationwide study in Australia to address these knowledge gaps by identifying key factors (patient- and healthcare-related) influencing healthcare pathways and management strategies after a traumatic knee injury. It will also explore the long-term patient-reported outcomes associated with different injury types and management strategies and examine potential disparities related to sex/gender, pre-injury activity level and access to care (eg, geographical remoteness, socioeconomic status).

To our knowledge, this is the first study to comprehensively map healthcare trajectories following acute knee injuries, encompassing both surgical and non-surgical management. The study design was developed with input from a multidisciplinary group of experienced clinicians and researchers, as well as a CAG comprising individuals with lived experience of acute knee injury. Consumer and community involvement led to several design decisions aimed at reducing participant burden, such as using short questionnaires and lay language instructions to simplify the reporting of healthcare pathways. As a result, the study employs single-item questions in some instances instead of full-length, validated patient-reported outcome measures (eg, using a single VAS item rather than the full Kessler Psychological Distress Scale for mental health, and only selected KOOS subscales are used). This approach prioritises feasibility, but we recognise that it may reduce the granularity and comprehensiveness of certain constructs, such as mental health. The fully online, self-reported design introduces potential recall bias, participant attrition and selection bias, as some patients with acute knee injuries may not be included.

## Supplementary material

10.1136/bmjsem-2025-002983 online supplemental file 1

## Data Availability

No data are available.
