# Supplementary material for: Traumatic knee injury healthcare pathways and outcomes: the Australian Knee Injury Inception Cohort Study (KIICS) protocol
Source: BMJ Open Sport Exerc Med. 2025 Nov 27;11(4):e002983. doi: 10.1136/bmjsem-2025-002983 (PMC12666194; doi:10.1136/bmjsem-2025-002983)
Supplement: online supplemental file 1 [file bmjsem-11-4-s001.docx]

**Supplementary Table 1.** Summary of outcome measures included in the Australian Knee Injury Inception Cohort Study

| **Construct / Outcome** | **Item / Question** | **PROM / Source** | **Psychometric Properties** |
| --- | --- | --- | --- |
| Patient satisfaction | “Taking into account your level of pain and function, if you were to remain for the next few months as you are today, would you consider that your current state is satisfactory?” (Yes/No) | PASS | Good content validity and good to excellent reliability (ICC = 0.8) in the context of knee injuries like ACL tears.^1,2^ |
| Health-related quality of life | EQ-5D-5L: 5 domains (mobility, self-care, usual activities, pain/discomfort, anxiety/depression) + index score; EQ-VAS: “How is your health today?” (0–100) | EQ-5D-5L / EQ-VAS | Extensively validated internationally and in Australia; widely used in knee injury registries.^3,4^ |
| Mental health impact | “In the last week, how has your knee injury impacted your mental health?” (VAS 0–100) | Study-specific single VAS | VAS validated for distress/depression^5^; adapted for knee injury context. |
| Knee pain | “In the last week, on average, how painful was your knee?” (VAS 0–100) | Study-specific single VAS | VAS widely validated for pain intensity; high reliability and responsiveness.^6^ |
| Knee instability | i) “During the last week… how stable is your knee in everyday situations?” (VAS 0–100); ii) “...how stable is your knee in rehab/sport activities?” (VAS 0–100) | Study-specific single VAS items | VAS validated for subjective construct like pain^6^; adapted for knee perceived instability context. |
| Activity level | Tegner Activity Scale (0–10; 0 = disability pension due to knee problems, 10 = elite national/international sport) | Tegner Activity Scale (TAS) | Valid, reliable, responsive in ACL and meniscal injury populations.^7^ |
| Global rating of change | “Overall, how would you rate your knee compared to when you first injured it?” (7-point Likert, 1 = very much worse, 7 = completely recovered) | Global Rating of Change scale | Widely used in musculoskeletal injury; good construct validity.^8^ |
| Function in sport/recreation | 4 items (KOOS-Sport/Rec subscale) | KOOS | Full questionnaire validated in young patients with a knee injury^9^; high reliability and responsiveness.^10^ |
| Knee-related quality of life | 4 items (KOOS-QoL subscale) | KOOS | Full questionnaire validated in young patients with a knee injury^9^; high reliability and responsiveness.^10^ |
| Fear of reinjury | “How fearful are you of re-injuring your knee?” (VAS 0–100) | Single item from ACL-QoL | Full questionnaire developed and validated in ACL populations; good construct validity.^11^ |

*PASS: Patient Acceptable Symptom State; ICC: Intraclass correlation coefficient; ACL: Anterior Cruciate Ligament; VAS: Visual Analog Scale; TAS: Tegner Activity Scale; KOOS: Knee injury and Osteoarthritis Outcome Score; QoL: Quality of life*

**References**

1. Muller B, Yabroudi MA, Lynch A, et al. Defining Thresholds for the Patient Acceptable Symptom State for the IKDC Subjective Knee Form and KOOS for Patients Who Underwent ACL Reconstruction. *Am J Sports Med*. 2016;44(11):2820-2826. doi:10.1177/0363546516652888

2. Tubach F, Ravaud P, Baron G, et al. Evaluation of clinically relevant states in patient reported outcomes in knee and hip osteoarthritis: the patient acceptable symptom state. *Ann Rheum Dis*. 2005;64(1):34-37. doi:10.1136/ard.2004.023028

3. Herdman M, Gudex C, Lloyd A, et al. Development and preliminary testing of the new five-level version of EQ-5D (EQ-5D-5L). *Qual Life Res*. 2011;20(10):1727-1736. doi:10.1007/s11136-011-9903-x

4. Norman R, Mulhern B, Lancsar E, et al. The Use of a Discrete Choice Experiment Including Both Duration and Dead for the Development of an EQ-5D-5L Value Set for Australia. *PharmacoEconomics*. 2023;41(4):427-438. doi:10.1007/s40273-023-01243-0

5. Huang Z, Kohler IV, Kämpfen F. A Single-Item Visual Analogue Scale (VAS) Measure for Assessing Depression Among College Students. *Community Ment Health J*. 2020;56(2):355-367. doi:10.1007/s10597-019-00469-7

6. Delgado DA, Lambert BS, Boutris N, et al. Validation of Digital Visual Analog Scale Pain Scoring With a Traditional Paper-based Visual Analog Scale in Adults. *J Am Acad Orthop Surg Glob Res Rev*. 2018;2(3):e088. doi:10.5435/JAAOSGlobal-D-17-00088

7. Briggs KK, Lysholm J, Tegner Y, Rodkey WG, Kocher MS, Steadman JR. The reliability, validity, and responsiveness of the Lysholm score and Tegner activity scale for anterior cruciate ligament injuries of the knee: 25 years later. *Am J Sports Med*. 2009;37(5):890-897. doi:10.1177/0363546508330143

8. Kamper SJ, Maher CG, Mackay G. Global rating of change scales: a review of strengths and weaknesses and considerations for design. *J Man Manip Ther*. 2009;17(3):163-170. doi:10.1179/jmt.2009.17.3.163

9. Roos EM, Lohmander LS. The Knee injury and Osteoarthritis Outcome Score (KOOS): from joint injury to osteoarthritis. *Health Qual Life Outcomes*. 2003;1:64. doi:10.1186/1477-7525-1-64

10. Collins NJ, Prinsen C a. C, Christensen R, Bartels EM, Terwee CB, Roos EM. Knee Injury and Osteoarthritis Outcome Score (KOOS): systematic review and meta-analysis of measurement properties. *Osteoarthritis Cartilage*. 2016;24(8):1317-1329. doi:10.1016/j.joca.2016.03.010

11. Mohtadi N. Development and validation of the quality of life outcome measure (questionnaire) for chronic anterior cruciate ligament deficiency. *Am J Sports Med*. 1998;26(3):350-359. doi:10.1177/03635465980260030201
